# Roles of type II thioesterases and their application for secondary metabolite yield improvement

**DOI:** 10.1007/s00253-014-5952-8

**Published:** 2014-08-02

**Authors:** Magdalena Kotowska, Krzysztof Pawlik

**Affiliations:** 1 Ludwik Hirszfeld Institute of Immunology and Experimental Therapy, Polish Academy of Sciences, ul. Rudolfa Weigla 12, 53-114 Wroclaw, Poland; 2 Department of Toxicology, Wroclaw Medical University, ul. Borowska 211, 50-552 Wroclaw, Poland

**Keywords:** Type II thioesterase, Polyketide synthase, Nonribosomal peptide synthetase, PKS, NRPS, Synthetic biology

## Abstract

A large number of antibiotics and other industrially important microbial secondary metabolites are synthesized by polyketide synthases (PKSs) and nonribosomal peptide synthetases (NRPSs). These multienzymatic complexes provide an enormous flexibility in formation of diverse chemical structures from simple substrates, such as carboxylic acids and amino acids. Modular PKSs and NRPSs, often referred to as megasynthases, have brought about a special interest due to the colinearity between enzymatic domains in the proteins working as an “assembly line” and the chain elongation and modification steps. Extensive efforts toward modified compound biosynthesis by changing organization of PKS and NRPS domains in a combinatorial manner laid good grounds for rational design of new structures and their controllable biosynthesis as proposed by the synthetic biology approach. Despite undeniable progress made in this field, the yield of such “unnatural” natural products is often not satisfactory. Here, we focus on type II thioesterases (TEIIs)—discrete hydrolytic enzymes often encoded within PKS and NRPS gene clusters which can be used to enhance product yield. We review diverse roles of TEIIs (removal of aberrant residues blocking the megasynthase, participation in substrate selection, intermediate, and product release) and discuss their application in new biosynthetic systems utilizing PKS and NRPS parts.

## Introduction

Microorganisms have been long exploited for their ability to produce antibiotics and a variety of other compounds of medical and industrial importance. The progress in sequencing genomes and metagenomes made in the last decade revealed the enormous potential for drug discovery by genome mining (Nett et al. 2009; Gao et al. 2010; Boddy 2014). Many species of actinomycetes have more than 20 gene clusters for secondary metabolites, out of which only a few products are known. The ways to get access to the new compounds include heterologous expression of whole gene clusters (Gao et al. 2010; Li and Neubauer 2014) and also construction of mutated or hybrid enzymatic complexes for production of modified structures (Baltz 2009; Olano et al. 2009a; Reeves and Rodriguez 2009). Synthetic biology has developed new concepts, such as “plug-and-play” engineering of predictable and controllable systems out of defined parts (biobricks) and “refactoring”—uncoupling them from native regulatory elements. Application of these approaches to biosynthesis of new bioactive compounds has become realistic with technological advances in de novo synthesis of hundred-kilobase long stretches of DNA covering whole gene clusters (Cummings et al. 2014; Yamanaka et al. 2014; Poust et al. 2014).

Although numerous examples of successful application of the above strategies for the production of “unnatural” natural compounds are available, obtaining high efficiency of such artificial systems remains a challenge (Li and Neubauer 2014; Poust et al. 2014). Here, we focus on an interesting class of enzymes—type II thioesterases (TEIIs) coded within secondary metabolite gene clusters—which may be used to improve product yield.

Modular polyketide synthases (PKSs) and nonribosomal peptide synthetases (NRPSs) are particularly well suited for combinatorial approach (Koglin and Walsh 2009; Reeves and Rodriguez 2009). Both types of megasynthases remind assembly lines where each cycle of chain elongation is performed by a dedicated module made of covalently linked enzymatic domains. All substrates and intermediates are bound to the enzyme by a thioester linkage with a 4′-phosphopantetheine (4′PP) arm of an acyl carrier protein (ACP) domain in a PKS or peptidyl carrier protein (PCP) domain in an NRPS. A thioesterase domain (type I thioesterase (TEI)) is usually located at the C-terminus of the last module and is responsible for the release of the full-length chain by hydrolysis and often macrolactonization (Staunton and Weissman 2001). In some cases, C-terminal reductase domains are found in place of TEI, and their role in reductive product release was shown or hypothesized (Gomez-Escribano et al. 2012 and references therein). Several examples of chain release by discrete hydrolytic enzymes were also described (Oliynyk et al. 2003; Liu et al. 2006, 2008; Xu et al. 2013; Zabala et al. 2014).

Many PKS and NRPS gene clusters contain genes for TEIIs—discrete, 25–29-kDa proteins belonging to the α/β hydrolase family which hydrolyze residues attached by thioester bonds to 4′PP arms of acyl or PCPs. They are called “type II” to stress the fact that they are not covalently bound to the multienzyme, as opposed to TE domains (TEIs). TEIIs are in most cases not essential for the synthesis of the corresponding synthase product but play an important role in maintaining the efficiency of the assembly line. In this article, the roles of TEIIs involved in polyketide and nonribosomal peptide biosynthesis, are reviewed and the implications for combinatorial/synthetic biology approaches are discussed.

## Functions of TEIIs

TEIIs were first identified in some vertebrate fatty acid synthase (FAS) complexes, where they play a role of alternative chain terminating enzymes. FAS TEIIs hydrolyze thioesters of shorter carbon chains (C8, C10, and C12) than the main product—palmitic acid (C16)—released by the TEI domain of FAS (Libertini and Smith 1978; Smith 1994).

**Fig. 1:**
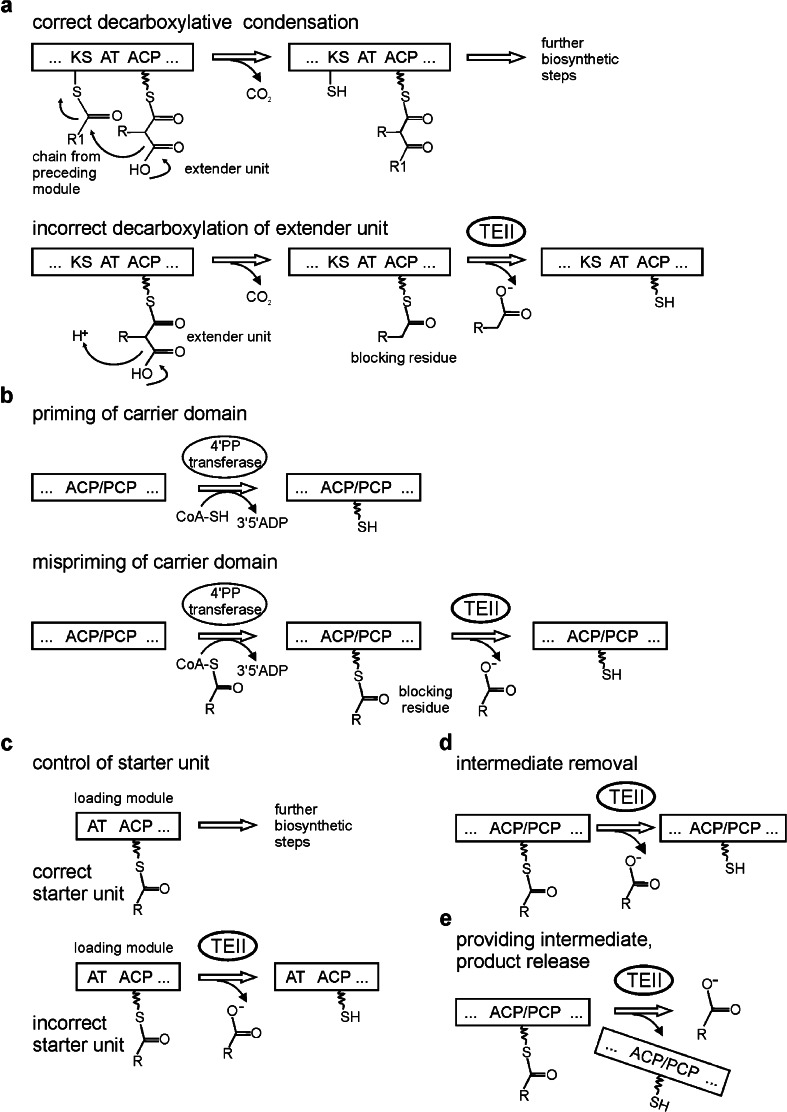
Roles of type II thioesterases (*TEIIs*) associated with polyketide synthases and nonribosomal peptide synthetases. *Wavy line* represents 4′-phosphopantetheine (*4′PP*). *KS* ketosynthase, *AT* acyltransferase, *ACP* acyl carrier protein, *PCP* peptidyl carrier protein. **a** Normal polyketide chain elongation starts with a decarboxylative condensation catalyzed by the KS domain. Incorrect decarboxylation of an extender unit without condensation gives rise to an acyl residue attached to the ACP. Editing TEII hydrolyzes the acyl residue and leaves the ACP free to accept a new, dicarboxylated extender unit transferred by the AT domain. **b** ACP and PCP domains are primed with a cofactor—4′PP—which is transferred by a 4′PP transferase from coenzyme A (*CoA*). 4′PP transferases can also accept acyl-CoA as a substrate and misprime carrier domains with acyl-4′PP, thus making the attachment site inaccessible for an extender unit. Editing TEII hydrolyzes the acyl residue and leaves the carrier domain free to accept a correct substrate. **c** TEII influences the choice of the starter unit by specific hydrolysis of some residues attached to the ACP of the loading module. The residue which is not cleaved becomes a starting unit for the polyketide chain. **d** Promiscuous TEIIs can remove amino acids and polyketide synthesis intermediates, including correct dicarboxylated extender units, if they are not readily processed by downstream domains. **e** TEIIs specific for certain moieties which are required for the construction of the full structure of the metabolite release them from specific carriers to make them available for the next biosynthetic step. Some TEIIs release complete PKS/NRPS products from the multienzyme

Gene disruption experiments and studies of the catalytic activity of TEII proteins against synthetic substrates showed that in PKS and NRPS systems, these enzymes have a corrective (editing) role, removing undesirable substrates and nonreactive moieties that block the NRPS and PKS. They can also take part in the selection of starter units to be incorporated by the synthases. In some cases, TEIIs are responsible for the release of intermediates or final products (Fig. 1).

Synthetic substrates used to examine the activity of TEIIs in  vitro include derivatives of coenzyme A (CoA), *p*-nitrophenyl esters (*p*-NP), and thioesters of *N*-acetylcysteamine (NAC) which allow spectrophotometric measurement of the hydrolysis progress. NAC is a structural analog of phosphopantetheine. Another group of substrates more closely resembling the natural systems are isolated ACP (PCP) domains or even entire PKS (NRPS) modules with different polyketide/peptide chains and short residues attached to 4′PP (Table 1).

**Table 1:** Enzymatic activity of type II thioesterases

Type II thioesterase	Substrate	k_cat_/K_M_ (M^−1^ s^−1^)	k_cat_ (min^−1^)	K_M_ (mM)	References
TylO, tylosin cluster of *Streptomyces fradiae* (PKS)	Acetyl-NAC	2.5	ND	ND	Heathcote et al. 2001
	Propionyl-NAC	12.9	29.2	37.9	
	Butyryl-NAC	6.5	11.0	28.2	
	Pentanoate-NAC	1.7	ND	ND	
	Diketide^a^-NAC	0.9	ND	ND	
	Acetyl-*p*-NP	83	ND	ND	
	Propionyl-*p*-NP	439	ND	ND	
	Butyryl-*p*-NP	306	ND	ND	
	Pentanoate-*p*-NP	284	ND	ND	
	Triketide^b^-*p*-NP	39	ND	ND	
	Acetyl-NAC	5.8	14.8	42.8	Zhou et al. 2008
	Propionyl-NAC	10.9	23.2	35.6	
FscTE, FR-008/candicidin cluster of *Streptomyces sp*. strain FR-008 (PKS)	Acetyl-NAC	35.7	71.6	33.4	Zhou et al. 2008
	Propionyl-NAC	56.9	109.2	32.0	
ScoT, coelimycin cluster of *Streptomyces coelicolor* A3(2) (PKS)	Acetyl-NAC	33	109	56	Kotowska et al. 2009
	Propionyl-NAC	221	450	34	
	Butyryl-NAC	17	54	52	
	Acetyl-*p*-NP	788	ND	ND	
	Propionyl-*p*-NP	3567	ND	ND	
	Butyryl-*p*-NP	485	ND	ND	
PikAV, pikromycin cluster of *Streptomyces venezuelae* (PKS)	Acetyl-AT_L_ACP_L_	4.9	ND	ND	Kim et al. 2002
	Propionyl-AT_L_ACP_L_	15.8	ND	ND	
	Butyryl-AT_L_ACP_L_	17.5	ND	ND	
	Malonyl-AT_L_ACP_L_	3.9	ND	ND	
	Methylmalonyl-AT_L_ACP_L_	3.3	ND	ND	
	Methylmalonyl-PikAIII	2.9	ND	ND	
NanE, nanchangmycin cluster of *Streptomyces nanchangensis* NS3226 (PKS)	Nanchangmycin-NAC	2.5	0.0036	0.024	Liu et al. 2008
	Nanchangmycin aglycone-NAC	0.15	0.0020	0.220	
	Monensin-NAC	0.070	0.00115	0.270	
	Salinomycin-NAC	−	−	−	
	Diketide^c^-NAC	0.080	0.210	44	
	Ketodiketide^d^-NAC	1.35	5.2	64	
	Seco-10-deoxymethynolide-NAC	−	−	−	
	Seco-7-dihydro-10-deoxymethynolide-NAC	−	−	−	
TycF, tyrocidine cluster of *Bacillus brevis* (NRPS)	Acetyl-NAC	2.2	ND	ND	Yeh et al. 2004
	Ala-NAC	1.8	ND	ND	
	Acetyl-D-Ala-NAC	1.0	ND	ND	
	Acetyl-D-Leu-NAC	5.0	ND	ND	
	D-Phe-NAC	6.8	ND	ND	
	Phe-NAC	5.3	ND	ND	
	Acetyl-Phe-NAC	7.2	ND	ND	
	Leu-NAC	4.0	ND	ND	
	Acetyl-Leu-NAC	23.3	ND	ND	
	D-Phe-PheATE	2500	ND	ND	
	Phe-PheATE	3000	ND	ND	
TEII_srf_, surfactin cluster of *Bacillus subtilis* (NRPS)	D-Phe-PheATE	3000	ND	ND	Yeh et al. 2004
	Phe-PheATE	2666	ND	ND	
	Acetyl-PCP	1.75 x 10^6^	95	0.9 x 10^-3^	Schwarzer et al. 2002
RifR, rifamycin cluster of *Amycolatopsis mediterranei* (PKS/NRPS)	Decanoyl-CoA	160	ND	ND	Claxton et al. 2009
	Octanoyl-CoA	31	ND	ND	
	Propionyl-CoA	25	ND	ND	
	Butyryl-CoA	13	ND	ND	
	Acetyl-CoA	11	ND	ND	
	Isobutyryl-CoA	9.6	ND	ND	
	Hexanoyl-CoA	5.9	ND	ND	
	Methylmalonyl-CoA	1.8	ND	ND	
	Malonyl-CoA	1.5	ND	ND	
	Propionyl-RifM1	210	ND	ND	
	Acetyl-RifM1	150	ND	ND	
	Methylmalonyl-RifM1	54	ND	ND	
RedJ, prodiginine cluster of *S. coelicolor* A3(2) (PKS/NRPS)	Decanoyl-RedQ	460	138	5.2	Whicher et al. 2011
	Decanoyl-AcpP	660	198	5.0	
	Dodecanoyl-AcpP	3316	498	2.5	
	Acetyl-AcpP	55	200	60	
	Malonyl-AcpP	−	−	−	
	Malonyl-RedQ	−	−	−	
	Decanoyl-CoA	0.67	3.0	70.0	
	Dodecanoyl-CoA	2.08	2.0	16.7	
	Acetyl-CoA	0.02	1.2	1050	
	Malonyl-CoA	−	−	−	
Type II thioesterase	Substrate	Activity			References
TEII, DEBS *cluster of Saccharopolyspora erythraea* (PKS)	Acetyl-ACP2	−			Hu et al. 2003
	Acetyl-ACP_L_	++			
	Propionyl-ACP_L_	−			
	Butyryl-ACP_L_	−			
ScoT, coelimycin cluster of *Streptomyces coelicolor* A3(2) (PKS)	Propionyl-*p*-NP	++			Kotowska et al. 2014
	Dodecanoyl-*p*-NP	+			
TEII_srf_, surfactin cluster of *Bacillus subtilis* (NRPS)	Pro-ProCAT	+			Schwarzer et al. 2002
	D-Phe-Pro-Leu-TycA/ProCAT-LeuCAT	+			
	Acetyl-ACP_FAS_	−			
TEII_bac_, bacitracin cluster of *Bacillus licheniformis* (NRPS)	Pro-ProCAT	+			
	Acetyl-PCP	++			
	Acetyl-ACP_FAS_	−			

Results of the experiments where kinetic constants were not determined are shown qualitatively in the second part of the table. Symbols and abbreviations:

++ high activity, + low activity, − no reaction, *CoA* coenzyme A, *NAC* *N*-acetyl cysteamine, *ND* not determined, *p-NP p*-nitrophenyl, *ACP2* ACP from module 2 of 6-deoxyerythronolide synthase (*DEBS*), *ACP*_*FAS*_ ACP from the fatty acid synthase, *ACP*_*L*_ ACP from the loading module of DEBS, *AcpP* ACP from *E. coli* fatty acid synthase, *AT*_*L*_ *ACP*_*L*_ loading module of DEBS, *PCP* PCP domain from module 2 of tyrocidine synthetase, *PheATE* module 1 of gramicidin synthetase,* PikAIII* module 5 of pikromycin synthase, *ProCAT* module 2 of tyrocidine synthetase, *RedQ* ACP of the prodiginine pathway, *RifM1* module 1 of rifamycin synthase, *TycA/ProCAT-LeuCAT* hybrid NRPS made of modules 1, 2 and 10 of tyrocidine synthetase

^a^Analog of the first condensation product with opposite configuration of hydroxyl group

^b^Analog of the second condensation product with opposite configuration of methyl group

^c^(2S,3R)-2-Methyl-3-hydroxypentanoyl-NAC

^d^2-Methyl-3-ketopentanoyl-NAC

Individual thioesterases differ significantly in their substrate specificities and activities. Exploring the mechanisms of action and kinetic parameters of TEIIs is important from the point of view of potential practical applications of these enzymes.

### Removal of nonreactive acyl residues

#### Origins of acyl residues blocking the megasynthases

Biosynthesis of polyketides involves repeated cycles of condensation and reduction of short carboxylic acids in a manner similar to fatty acid biosynthesis (Staunton and Weissman 2001). The first step of each biosynthetic cycle is a decarboxylative condensation of a starter unit or the growing chain from the previous module with a dicarboxylic extender unit (in most cases, malonyl or methylmalonyl residue) attached to the ACP via a thioester bond with 4′PP (Fig. 1a). After reduction of the newly generated β-carbonyl group (depending on the presence of reducing domains in a given module), the chain is transferred to the subsequent module for the next elongation cycle. Occasionally, decarboxylation of extender units (mostly malonyl, methylmalonyl, and ethylmalonyl) occurs without condensation. Products of such aberrant decarboxylation (acetyl, propionyl, and butyryl residues, respectively) remain covalently attached to the ACP and block the assembly line (Fig. 1a) (Heathcote et al. 2001).

In the process of nonribosomal peptide synthesis, extender units are amino acids, and decarboxylation does not take place here. Schwarzer et al. (2002) identified a different mechanism of blocking NRPSs by nonreactive acyl residues. To obtain the activity, the PCP domain must have a covalently attached 4′PP. 4′PP transferase is responsible for priming the PCP domain with the cofactor. The source of 4′PP for the transferase is CoA, but the enzyme can also use different acylated derivatives of CoA (Fig. 1b) (Dall’Aglio et al. 2011). Indeed, it is often the case, since most of the CoA molecules in the cell are in the acylated form (Vallari et al. 1987). In the case of joining acyl-4′PP to PCP domain (mispriming), the binding site of amino acids and peptides is blocked, and this results in the inactivation of the NRPS complex prior to the first round of peptide synthesis. The acyl residue must be removed before the PCP domain can fulfill its function.

ACP domains of PKSs, similarly to the NRPS PCP domains, require the 4′PP cofactor and are turned to holoenzyme form by the respective 4′PP transferases. It is very likely that, in addition to erroneous decarboxylation of extender units, mispriming of ACP with acyl-4′PP results in blocking of the PKS with nonreactive residues (Pfeifer et al. 2002).

#### TEIIs with editing function

Modular megasynthases are usually built of several high molecular mass proteins (several hundreds of kilodaltons), and their synthesis and degradation are a significant effort for the cell. Clearly, restoring the performance of NRPSs and PKSs blocked by nonreactive residues would help to save resources. TEII is an important element of such a mechanism. As shown by biochemical studies, TEIIs hydrolyze acyl residues which may arise from aberrant decarboxylation of extender units or from mispriming of carrier domains with acyl-4′PP (Fig. 1a, b, Table 1) (Heathcote et al. 2001; Kim et al 2002; Schwarzer et al. 2002; Yeh et al. 2004; Zhou et al. 2008; Kotowska et al 2009).

It was shown that TEII (TylO) from tylosin synthase of *Streptomyces fradiae* hydrolyzes all three residues (acetyl, propionyl, and butyryl) that can be formed by aberrant decarboxylation of extender units incorporated by tylosin synthase (Table 1) (Heathcote et al. 2001). These observations are consistent with the results of the experiment, where inactivation of *tylO* gene reduced polyketide production to about 10–15 % of the normal level. Complementation of the mutation with a copy of *tylO* gene restored wild-type production level of polyketide (Butler et al. 1999). TEIIs from other PKSs are also capable of improving performance of a PKS lacking its own TEII. Complementation of the *tylO* mutation with *nmbB* gene from narbomycin synthase of *Streptomyces narbonensis* (Butler et al. 1999) and *scoT* gene from Cpk PKS of *Streptomyces coelicolor* A3(2) (Kotowska et al. 2002) increased polyketide production to, respectively, 35–40 and 48 % of the wild-type strain.

Deletion of *rifR* gene encoding TEII from rifamycin B hybrid PKS/NRPS of *Amycolatopsis mediterranei* lowered production of this compound to 40–60 % of normal levels (Doi-Katayama et al. 2000). RifR thioesterase has broad substrate specificity, hydrolyzing short, medium, and branched chain substrates (Table 1). It preferentially hydrolyzes decarboxylated acyl thioesters over natural (carboxylated) extender units, which is consistent with its editing role in removing blocking residues (Claxton et al. 2009).

Editing role of FscTE TEII associated with a modular PKS synthesizing FR008/candicidin was also confirmed by genetic and biochemical experiments (Zhou et al. 2008). TEII gene deletion reduced polyketide production by 90 %, which was restored by complementation with both native *fscTE* and heterologous *tylO* genes. Furthermore, it was shown that TEIIs originating from NRPS systems (tyrocidine and surfactin synthetases) were not able to complement the *fscTE* deletion (Zhou et al. 2008), reflecting their ability to distinguish between different ACP and PCP domains (Table 1) (Schwarzer et al. 2002; Yeh et al. 2004).

Hydrolytic activity of the recombinant TEII_srf_ protein from surfactin synthetase toward acetyl group linked to the isolated PCP domain is in accordance with the proposed function of the TEII as a scavenger of acyl residues blocking the NRPS (Table 1) (Schwarzer et al. 2002). Deletion of a gene encoding TEII_srf_ led to decrease of production by 84 % (Schneider and Marahiel 1998).

Similarly, *ybtT* thioesterase gene deletion strongly reduced the production of yersiniabactin siderophore by *Yersinia pestis*. Geoffroy et al. (2000) and Miller et al. (2010) reported that deletion mutant produced less than 6 % and approx. 25 % of wild-type level, respectively. On the other hand, synthesis of yersiniabactin in  vitro was achieved using four biosynthetic proteins, without TEII. Addition of YbtT thioesterase to the reaction mixture did not increase the biosynthesis rate (Miller et al. 2002). However, in the in  vitro experiment, only necessary precursors were present. It is likely that in  vivo, aberrant substrates interfere with the normal process and the editing thiesterase is needed to remove them. Successful heterologous production of yersiniabactin was obtained when the hybrid PKS/NRPS was expressed in *Escherichia coli*. Although the presence of TEII was found not to be necessary in this host, yersiniabactin production increased 2.5 times upon expression of *ybtT* (Pfeifer et al. 2003).

Surprisingly, inactivation of ScoT thioesterase from Cpk synthase which had been shown earlier to act as an editing thiesterase in a heterologous system (Kotowska et al. 2002) resulted in complete loss of coelimycin production (Kotowska et al. 2014). Such a strong effect of TEII inactivation is typical of chain terminating enzymes (see below); however, in the case of ScoT, this is unlikely, as the recombinant protein has very weak hydrolytic activity toward a 12-carbon chain mimicking the polyketide chain as opposed to short acyl residues (Table 1) (Kotowska et al. 2009, 2014). Polyketide precursor of coelimycin is probably released by a reductase domain (Gomez-Escribano et al. 2012).

It is not clear why the removal of editing thioesterases is detrimental to the efficiency of some megasynthases, and it apparently does not affect others. For example, deletion of *pikAV* gene coding TEII from the picromycin/methymycin synthase (Pik PKS) of *Streptomyces venezuelae* had no influence on the level of polyketide production (Chen et al. 2001). However, its coexpression with Pik PKS in a heterologous host (*Streptomyces lividans*) resulted in twofold to sevenfold increase in polyketide production levels (Tang et al. 1999). Perhaps, its effect in the native host is significant only in special circumstances other than the conditions of the experiment optimized for antibiotic production. It is noteworthy that overexpression of *pikAV* gene in *S. venezuelae* resulted in 50–70 % reduction of polyketide production (Kim et al. 2002), which can be explained by removal of correct substrates from the synthase. PikAV TEII was shown to hydrolyze malonyl and methylomalonyl chains representing correct extender units attached to ACP at a similar rate as acyl groups representing nonreactive residues (Table 1) (Kim et al. 2002).

### Removal of aberrant intermediates

Activity of TylO thioesterase was also tested against a diketide and triketide residues representing abnormal intermediates of tylosin biosynthesis (Table 1) (Heathcote et al. 2001). Such substrates were hydrolyzed with much lower efficiency than short acyl groups; hence, the conclusion that they are not significant TEII substrates in vivo. Nevertheless, they can be hydrolyzed if they remain exposed for a sufficiently long time (Fig. 1d). Acyltransferase domains recruiting extender units in PKSs have high specificity; however, as shown by the practice of combinatorial biosynthesis and modified substrates feeding, the remaining domains, including the terminal thioesterase, accept a wide range of modified molecules (McDaniel et al. 1999; Cummings et al. 2014). Therefore, potentially erroneous intermediates should not be the cause of blocking the PKS assembly line; they would rather contribute to the formation of by-products.

Although adenylating domains of NRPSs are highly specific, they occasionally incorporate amino acids that are not accepted by the condensing domain, which results in stalling the synthesis process. Therefore, the editing role of TEIIs appears to include the removal of amino acids and peptides that block the NRPS, in addition to hydrolysis of acyl groups arising due to mispriming. TEIIs from surfactin and bacitracin synthetases hydrolyzed PCP-bound intermediates which could not be further processed by the NRPS (amino acid and tripeptide), however, much more slowly than the acetyl group (Table 1) (Schwarzer et al. 2002). Tioesterase TycF from *Bacillus brevis* showed no activity on the correct intermediates of tyrocidine synthesis (two to five amino acid chains), but individual amino acids were hydrolyzed with a yield comparable to that of the acetyl group (Table 1) (Yeh et al. 2004).

### Control of starter units

Propionate is the correct starter unit for 6-deoxyerythronolide B (6dEB) synthesis—the erythromycin precursor produced by the most investigated modular type I PKS—DEBS of *Saccharopolyspora erythraea.* Inactivation of the gene encoding the TEII caused a significant admixture of 15-norerytromycin, which is a derivative of 15-nor-6dEB resulting from the incorporation of acetate instead of propionate as a starter unit. There was no significant decrease in the total production of polyketides, it remained at 80 % of the normal level (Hu et al. 2003). An experiment was conducted on the DEBS TEII activity against acetyl, propionyl, and butyryl residues attached to isolated ACP domains from the DEBS loading module (ACP_L_) and from module 2 of DEBS (ACP_2_). TEII was active only against the acetyl group bound to ACP_L_ (Table 1) (Hu et al. 2003). This led to the conclusion that the TEII is involved in the control of the starter unit by the loading module (Fig. 1c).

Brown et al. (1995) observed previously that the major product of the first subunit of the synthase (DEBS1) expressed in *S.  coelicolor* A3(2) was the nor-analog derived from acetate as a starter unit instead of propionate. This can be explained by the lack of the TEII selectively removing acetate from ACP_L_ and potentially by strong preference for propionate of TEII ScoT from *S.  coelicolor* Cpk PKS (Kotowska et al. 2009).

When DEBS genes were expressed in *S. lividans* and *S. coelicolor* A3(2), it was observed that coexpression of TEII significantly reduced the content of 15-nor-6dEB and also caused a twofold increase of concentration of products (Hu et al. 2003). Similarly, upon coexpression of DEBS genes with TEII in *E. coli*, the production of 6dEB has doubled. Interestingly, in *E. coli*, no 15-nor-6dEB was detected even in the absence of TEII (Pfeifer et al. 2002; Hu et al. 2003). In experiments, in which improper acylation of ACP_L_ of DEBS was avoided by inactivation of ketosynthase domain of module 1 and feeding to the culture of a correct diketide accepted directly by module 2, addition of TEII also increased twice the level of produced polyketide (Hu et al. 2003). This indicates that the positive effect of TEII on DEBS performance is not restricted to the control of the starter unit.

Other examples of starter unit control by TEIIs come from nonmodular PKSs. Type II PKS systems producing nonacetate-primed polyketides include proteins homologous to FabD (malonyl-ACP/CoA transferase) which were first postulated to load the unusual starters on the corresponding ACPs. It was found later that those FabD homologues, such as ZhuC from R1128 PKS (Tang et al. 2004), EncL from enterocin PKS (Kalaitzis et al. 2011), and presumably DpsD from daunorubicin PKS (Castaldo et al. 2008), are in fact thioesterases which facilitate priming of ACP with the correct starter units (butyrate, benzoate, and propionate, respectively) by hydrolyzing “unwanted” acetyl residues (Fig. 1c).

TEII AsuC15 from asukamycin biosynthetic cluster also influences the starter unit selection for polyketide chain, but in a different manner—maintaining balance between different compounds and preventing any of them from becoming dominant. Asukamycin, a mixture of related antimicrobial and potential antitumor compounds isolated from *Streptomyces nodosus* subsp. *asukaensis*, is built from two triene chains with unusual starter units assembled by type II and III PKSs (Rui et al. 2010). So-called lower polyketide chain starts with 3-amino-4-hydroxybenzoic acid which then becomes a core structure common to all asukamycins. The upper chain starts with residues which are also substrates for FAS: cyclohexylcarbonyl-CoA (CHC-CoA) in asukamycin A1 and branched acyl-CoA in related compounds. Besides, CHC-CoA is a precursor of ω-cyclohexyl fatty acids which account for 3.1 % of total cellular fatty acids, and branched chains are predominantly used for fatty acid synthesis in *Streptomyces*.

AsuC15 can remove the CHC-acyl intermediate from AsuC5-ACP to enhance the production of branched starter-derived compounds, as the *asuC15* mutant predominantly accumulates asukamycin A1 rather than an equal amount of all the variants observed in the wild-type strain. AsuC15 thioesterase is also proposed to suppress excess ω-cyclohexyl fatty acid formation and help to maintain membrane homeostasis by releasing the CHC residue from FAS ACP, as the percentage of ω-cyclohexyl fatty acids was increased fivefold in the *asuC15* mutant (Rui et al. 2010). The double role of AsuC15 TEII is an interesting example of primary and secondary metabolism interconnection.

### Providing key intermediates

Another role for TEIIs found in secondary metabolite biosynthetic complexes is the release of pathway intermediates from a carrier domain allowing them to be further processed by subsequent proteins (Fig. 1e). This can be exemplified by the synthesis of kutznerides, a mixture of nine antimicrobial cyclic hexadepsipeptides containing 2-(1-methylcyclopropyl)-d-glycine (MecPGly), isolated from the soil actinomycete *Kutzneria* sp. 744 (Fujimori et al. 2007). The kutzneride cluster encodes three multidomain proteins, which make up six NRPS modules and four stand-alone proteins: KtzB and KtzN which are homologous to adenylation domains, KtzC homologous to a thiolation domain (PCP) and KtzF—a TEII. The KtzB, N, and C proteins are proposed to form a “module” synthesizing the MecPGly moiety, which is then released by KtzF thioesterase and loaded on the next NRPS protein.

In nikkomycin antibiotics biosynthesis, the β-hydroxy-His is released from the didomain NRPS NikP1 by a TEII NikP2 and is further transformed to the imidazolone base found in nikkomycins X/I (Chen et al. 2002). When NikP2 was disrupted in *Streptomyces tendae* Tü901, the mutant strain failed to produce nikkomycins X/I. The disruption did not affect production of nikkomycins Z/J in which the imidazolone base is replaced by uracil base (Lauer et al. 2001). A similar thioesterase Bph from *Amycolatopsis balhimycina* releases from a carrier protein β-hydroxy-Tyr—a nonproteinogenic amino acid which is incorporated into the backbone of a glycopeptide antibiotic balhimycin (Mulyani et al. 2010). Inactivation of Bph resulted in complete block of balhimycin synthesis which could be restored by addition of free β-hydroxy-Tyr (Puk et al. 2002).

Prodiginines are a class of bioactive products of *S. coelicolor* A3(2) which are synthesized by a hybrid PKS/NRPS system (*red* gene cluster). Products of *red* genes act in concert with the primary metabolism enzymes from fatty acid synthesis which assemble a dodecanoyl chain attached by a thioester bond to RedQ (ACP protein coded within the *red* cluster). The TEII RedJ was shown to facilitate transfer of the dodecanoyl group from RedQ onto a multidomain protein RedL which performs further elongation (Whicher et al. 2011). Deletion of RedJ gene leads to a 75 % decrease in prodiginine production. Biochemical studies of enzymatic activity of RedJ proteins showed its strong preference for dodecanoyl residue over short chains and selectivity toward RedQ over FAS ACP (Whicher et al. 2011).

### Releasing products

Polyether ionophore antibiotics such as monensin, nanchangmycin, and nigericin are synthesized by modular PKSs which lack terminal TE domains (Liu et al. 2009). The polyunsaturated polyketide chain undergoes a cascade of oxidative cyclizations being still attached to the ACP, as opposed to classic macrolide polyketides which are released by a TEI domain and then are subject to post-polyketide modifications. It was shown recently that the intermediate polyketide chain is transferred from the modular PKS to a discrete ACP protein which serves as a carrier for further steps of polyether formation (Harvey et al. 2007). The final product is then hydrolyzed by a TEII (Fig. 1e).

Monensin and nanchangmycin are released by TEIIs which were originally assigned as epoxide hydrolases: MonCII and NanE, respectively (Harvey et al. 2006; Liu et al. 2006, 2008). NigCII is proposed to play a similar role in nigericin biosynthesis, based on its more than 50 % sequence similarity to NanE and MonCII. Deletion of *monCII* and *nanE* genes resulted in complete inhibition of respective polyketides production (Oliynyk et al. 2003; Liu et al. 2006). Recombinant MonCII and NanE proteins preferentially hydrolyzed NAC derivatives of monensin and nanchangmycin, respectively, over substrates representing intermediates of earlier steps of biosynthesis (Harvey et al. 2006; Liu et al. 2006, 2008). Monensin PKS cluster of *Streptomyces cinnamonensis* contains two other genes coding TEIIs, namely *monAIX* and *monAX* which are postulated to have an editing role. In concordance with that, deletion of these genes caused only a modest drop in monensin yield, and MonAIX was found to efficiently hydrolyze acetate residue (Harvey et al. 2006).

An experiment, which demonstrated the importance of a TEII for a hybrid PKS/NRPS efficiency, was the inactivation of the TEII gene from *Streptomyces lydicus* AS 4.2501, resulting in an almost complete loss of production of RNA polymerase inhibitor streptolydigin (Yu et al. 2006). Its close homologue SlgB from *S.  lydicus* NRRL2433 was hypothesized to have an editing role (Olano et al. 2009b). Streptolydigin biosynthesis pathway was deciphered (Olano et al. 2009b; Gómez et al. 2012), but the authors did not propose the mode of product release from PKS/NRPS proteins (all multienzymes lacking the thioesterase or reductase domains). In our opinion, TEII is a likely candidate for a chain-releasing enzyme in streptolydigin biosynthesis.

Fungal type I iterative highly reducing PKSs are accompanied by TEIIs which release polyketide chains of desired length (Xu et al. 2013; Zabala et al. 2014). LovG TEII from lovastatin biosynthetic cluster of *Aspergillus terreus* was shown to release dihydromonacolin L acid from PKS protein and also play a role in removal of aberrant intermediates. Disruption of *lovG* gene dramatically decreased lovastatin production by *A.  terreus*. Interestingly, a small amount (less than 5 %) was still produced, since LovG could be replaced, with much smaller efficiency, by an endogenous *A.  terreus* thioesterase (Xu et al. 2013). In a detailed study on brefeldin A biosynthesis, Zabala et al. (2014) have shown recently that a TEII (Bref-TH thiohydrolase) from *Eupenicillium brefeldianum* ATCC 58665 releases an octaketide product from Bref-PKS leading to brefeldin A. Bref-PKS alone synthesized in  vitro longer products (nonaketides) which could be released by base hydrolysis. Chain length control by the releasing enzyme was also observed in fumagillin pathway by Zabala et al. (2014).

## Importance of TEIIs in synthetic biology approach to PKS- and NRPS-derived biosynthetic systems

Although constructing biological systems for production of novel biologically active compounds with designed chemical structures has become a reality, it is far from being applicable on an industrial scale. Modular multienzymes such as PKSs and NRPSs, including those known only from sequence analysis, offer a broad choice of potential parts “building bricks” to construct machineries to assemble unnatural secondary metabolites. Realization of this concept encounters a number of obstacles beginning with a shortage of ideas for really new chemical structures which could become better drugs. Therefore, analogs of existing compounds are usually tested.

Other aspects which need to be taken into account are the availability of precursors in the chosen host organism, substrate selectivity of chain assembling units, and compatibility of the parts of the machinery. Intermediates must be accepted by downstream domains/enzymes, including those responsible for chain release and further modifications such as glycosylation, methylation, and halogenation (Cummings et al. 2014; Poust et al. 2014).

As shown in the current review, overall performance of many megasynthases strongly depends on the presence of TEIIs. It is straighforward that TEIIs responsible for intermediate or product release are indispensable. It is not clear why some megasynthases must be accompanied by editing thioesterases, while others are not affected by TEII inactivation. One might speculate that variations in the ratio of free versus acylated CoA in the cell and in the specificity of 4′PP transferases may be responsible for differences in the frequency of ACP (PCP) mispriming. In some PKS and NRPS clusters, no TEII genes were identified, and in others, multiple TEII genes were found, where they probably have their specialized functions. In general, coexpression of native or heterologous TEIIs increased the level of production of final compounds. However, an adverse effect of excess of a TEII due to correct substrates removal has also been reported (Kim et al. 2002).

We suggest that inclusion of an appropriate TEII should be carefully considered when designing an artificial system built from PKS and NRPS parts. The features to analyze should include substrate specificity (in terms of both acyl or peptide residues and carrier domains) and the rate of hydrolysis. Potentially, TEIIs may be used to influence the starter unit selection. Chain length control by a discrete releasing enzyme appears to be a common feature of highly reducing iterative PKSs and could be exploited for generation of new compounds. In addition, it is possible that the activity of host TEIIs may interfere with the heterologous system under study.

Two models of action of TEIIs have been proposed (Heathcote et al. 2001; Claxton et al. 2009). In high specificity model, a TEII recognizes and hydrolyzes only aberrant residues, while in low specificity model, a TEII hydrolyzes with low rate both correct extender units and incorrect residues. Correct extender units are quickly processed by the PKS (NRPS), while residues stalling the ACP (PCP) domain for a sufficiently long time are removed even by a slow enzyme with low specificity. Currently, it is not possible to predict in detail the specificity of TEIIs on the basis of sequence analysis, and they must be studied experimentally case by case.

Structural data are available for three TEIIs: RifR from rifamycin hybrid PKS/NRPS (Claxton et al. 2009), RedJ from prodiginine hybrid PKS/NRPS (Whicher et al. 2011), and SrfD from surfactin NRPS (Koglin et al. 2008). It was found that TEIIs can have several conformations, and their conformational changes enable interactions with multienzyme domains. They possess flexible lid structures which change the size and shape of the active site chamber and control the access of substrates. For detailed discussions on the implications for substrate accommodation by TEIIs and comparison with TEI structures, see the comprehensive experimental articles cited above.

A new classification of all thioesterases has been proposed based on comparison of primary and tertiary structures and is available in a continuously updated database, ThYme (Thioester-active enzYmes, http://www.enzyme.cbirc.iastate.edu) that covers also other enzyme groups taking part in fatty acid synthesis (Cantu et al. 2010). According to this classification, TEIIs reviewed in this work belong to the family TE18 and thioesterase domains of PKSs, and NRPSs belong to the families TE16 (which includes also FAS TE domains) and TE17. A common feature of all three of them is the α/β hydrolase fold. There is also a small family TE15 which comprises a few hotdog fold thioesterases involved in polyketide synthesis. The role of two of them as chain terminating enzymes in enedyine synthesis was shown experimentally (Kotaka et al. 2009; Liew et al. 2010).

From the large number of identified TEIIs, only a few have been characterized in detail. In our opinion, these enzymes are worth further investigation, and their inclusion in a set of biobricks designed to build new biosynthetic machineries involving PKS/NRPS parts will be helpful in fine tuning of the systems.
